# Assessment of Risk for Sudden Cardiac Death Among Adolescents and Young Adults After Receipt of COVID-19 Vaccine — Oregon, June 2021–December 2022

**DOI:** 10.15585/mmwr.mm7314a5

**Published:** 2024-04-11

**Authors:** Juventila Liko, Paul R. Cieslak

**Affiliations:** 1 Public Health Division, Oregon Health Authority, Portland, Oregon

**Summary**

**What is already known about this topic?**

In April 2021, cases of myocarditis after COVID-19 vaccination, particularly among young male vaccine recipients, were reported to the Vaccine Adverse Event Reporting System.

**What is added by this report?**

To determine risk for sudden cardiac death among adolescents and young adults after COVID-19 vaccination, investigators examined June 2021–December 2022 Oregon death certificate data for decedents aged 16–30 years. Of 40 deaths that occurred among persons who had received an mRNA COVID-19 vaccine dose, three occurred ≤100 days after vaccination. Among these, two occurred in persons with underlying illness, and one decedent had an undetermined cause of death.

**What are the implications for public health practice?**

The data do not support an association of COVID-19 vaccination with sudden cardiac death among previously healthy young persons. COVID-19 vaccination is recommended for all persons aged ≥6 months to prevent COVID-19 and complications, including death.

## Abstract

COVID-19 vaccination has been associated with myocarditis in adolescents and young adults, and concerns have been raised about possible vaccine-related cardiac fatalities in this age group. In April 2021, cases of myocarditis after COVID-19 vaccination, particularly among young male vaccine recipients, were reported to the Vaccine Adverse Event Reporting System. To assess this possibility, investigators searched death certificates for Oregon residents aged 16–30 years who died during June 2021–December 2022 for cardiac or undetermined causes of death. For identified decedents, records in Oregon’s immunization information system were reviewed for documentation of mRNA COVID-19 vaccination received ≤100 days before death. Among 1,292 identified deaths, COVID-19 was cited as the cause for 30. For 101 others, a cardiac cause of death could not be excluded; among these decedents, immunization information system records were available for 88, three of whom had received an mRNA COVID-19 vaccination within 100 days of death. Of 40 deaths that occurred among persons who had received an mRNA COVID-19 vaccine dose, three occurred ≤100 days after vaccination. Two of these deaths were attributed to chronic underlying conditions; the cause was undetermined for one. No death certificate attributed death to vaccination. These data do not support an association between receipt of mRNA COVID-19 vaccine and sudden cardiac death among previously healthy young persons. COVID-19 vaccination is recommended for all persons aged ≥6 months to prevent COVID-19 and complications, including death.

## Introduction

In December 2020, the Food and Drug Administration authorized two COVID-19 mRNA vaccines for use in the United States. Early vaccine supplies were prioritized for health care personnel and long-term care facility residents, with phased vaccination of other persons, beginning with those who were older or had high-risk medical conditions, and concluding with healthy younger persons (*1*). In Oregon, healthy persons aged ≥16 years became eligible for COVID-19 vaccination on April 19, 2021. In April 2021, reports of myocarditis after COVID-19 vaccination, particularly among young male vaccine recipients, began to appear.*^,^^†^ Investigators in Israel estimated that the risk for myocarditis associated with receipt of mRNA COVID-19 vaccine was 2.13 per 100,000 among vaccine recipients, and was highest among adolescents and young adult males (10.69 per 100,000) (*2*). Published accounts suggest that postvaccination myocarditis is typically mild and associated with good outcomes after brief hospitalization (*3*,*4*). As of July 17, 2023, no fatal cases of myocarditis in Oregon had been reported to the federal Vaccine Adverse Event Reporting System (VAERS); however, because VAERS is a passive reporting system, adverse events after vaccination are likely underestimated. In late 2022, reports of sudden deaths among previously healthy young athletes, with suggested attribution to COVID-19 vaccination, appeared in the lay press^§^ and then in the medical literature (*5*,*6*). To ascertain whether young persons in Oregon might be dying from cardiac causes shortly after having received a COVID-19 vaccine dose, Oregon death certificate data were reviewed. 

## Methods

### Data Sources

Oregon law requires that a certificate of death be completed for each death in Oregon. Oregon’s vital records system abides by CDC’s National Center for Health Statistics’ data-quality standards^¶^, including extensive quality-assurance review. An independent source of data for assessing the completeness of death certificate reporting is not available. Data on Oregon resident deaths occurring outside the state are also collected through interstate exchange agreements. The ALERT Immunization Information System (IIS) is Oregon's statewide and lifespan immunization registry. During the COVID-19 pandemic, reporting of all COVID-19 vaccinations to ALERT IIS was mandated in Oregon.

### Data Analysis

To ascertain the occurrence of sudden cardiac deaths among adolescents or young adults that might plausibly be attributed to recent COVID-19 vaccination, investigators searched the Oregon death certificate database to identify persons aged 16–30 years who died during June 1, 2021–December 31, 2022 with “sudden death,” “arrhythmia,” “dysrhythmia,” “asystole,” “cardiac arrest,” “myocarditis,” “congestive heart failure,” “unknown,” “undetermined,” or “pending” cited among the immediate or four possible entries for underlying causes of death and other significant conditions contributing to death. Among the subset of decedents for whom death from a cardiac cause could not be ruled out by accompanying information in the death certificate database, records of mRNA COVID-19 vaccination within 100 days (*7*) before the date of death were retrieved from ALERT(IIS. Findings were stratified by sex. This activity was reviewed by the Oregon Health Authority, deemed not research, and was conducted consistent with applicable federal law and Oregon Health Authority policy.**

## Results

In Oregon, during June 2021–December 2022, a total of 1,292 deaths among persons aged 16–30 years were identified. These decedents included 925 (72%) males and 367 (28%) females (Figure).

**FIGURE:**
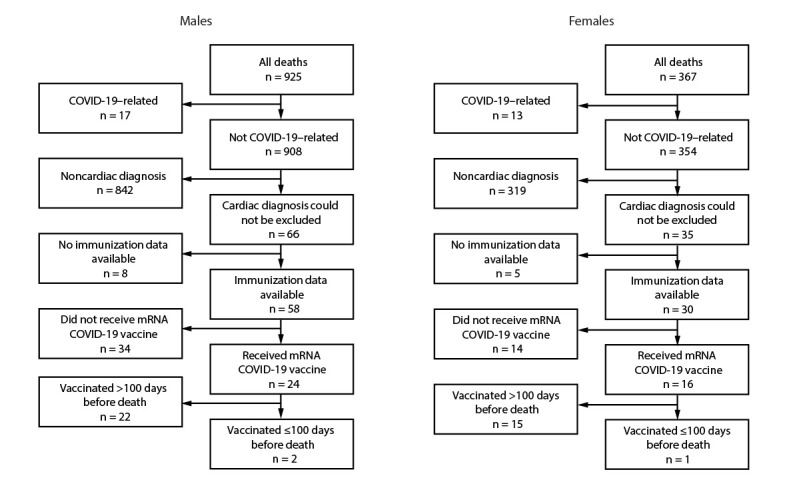
Deaths* among persons aged 16–30 years, by sex, cause of death,^†^ and mRNA COVID-19 vaccination status^§,¶,^** (N = 1,292) — Oregon, June 2021–December 2022 * Coded on the death certificate as sudden death, arrhythmia, dysrhythmia, asystole, cardiac arrest, myocarditis, congestive heart failure, unknown, undetermined, or pending. ^†^ Cardiac versus noncardiac. ^§^ Six of the 34 males who did not receive mRNA COVID-19 vaccine received Janssen (Johnson & Johnson) vaccine. ^¶^ An alternative plausible cause of death was identified for one of the males who had been vaccinated ≤100 days before death. After review of death certificate and medical examiner findings, an adverse event from COVID‐19 vaccination could neither be confirmed nor excluded as the cause for the other decedent. ** The only female decedent vaccinated ≤100 days before death was vaccinated 4 days before death. The manner of death was recorded as natural, and the immediate cause was “undetermined” as a consequence of chronic respiratory failure with hypoxia due to mitral stenosis.

### Male Decedents

Among the 925 male decedents, no death certificate listed vaccination either as the immediate or as a contributing cause of death. Overall, 17 (2%) deaths among males were attributed to COVID-19. Death certificates cited noncardiac causes of death or other conditions contributing to death for 842 (91%) of the male decedents. Among the remaining 66 (7%) male decedents, excluding a cardiac cause of death based on the death certificate was not possible. Among these 66 decedents, IIS vaccination records were available for 58 (88%); receipt of at least one mRNA COVID-19 vaccination was recorded for 24 (41%).

Among the 24 male decedents with an mRNA COVID-19 vaccination record in IIS, two (8%) died within 100 days of having received the vaccine. The first death was recorded as having occurred in a natural manner 21 days after COVID-19 vaccination. The immediate cause of death noted on the death certificate was congestive heart failure attributed to hypertension; other significant conditions included morbid obesity, type 2 diabetes, and obstructive sleep apnea. The second decedent had received a COVID-19 vaccine dose 45 days before the date of death; the cause of death was recorded as “undetermined natural cause.” Toxicology results were negative for alcohol, cannabinoids, methamphetamine, and opiates; aripiprazole, ritalinic acid, and trazodone were detected. Follow-up with the medical examiner could neither confirm nor exclude a vaccine-associated adverse event as a cause of death for this decedent.

### Female Decedents

Among the 367 female decedents, no death certificate listed vaccination as either the immediate or a contributing cause of death. Thirteen (4%) deaths were attributed to COVID-19. Noncardiac causes were recorded on the death certificates for 319 (87%) decedents. Among the remaining 35 (10%) female decedents, IIS records for 30 (86%) were identified, 16 (53%) of whom had documentation of receipt of at least 1 mRNA COVID-19 vaccine dose. Only one of these deaths occurred within 100 days of having received an mRNA COVID-19 vaccine dose; the decedent died 4 days after COVID-19 vaccination. The manner of death was recorded as natural, and the immediate cause was listed as undetermined but as a consequence of chronic respiratory failure with hypoxia attributed to mitral stenosis.

## Discussion

Electronic health record data from 40 U.S. health care systems during January 2021–January 2022, showed that the risk for cardiac complications was significantly higher after COVID-19 infection than after mRNA COVID-19 vaccination among persons aged ≥5 years (*8*). Data from CDC’s National Center for Health Statistics show a background mortality rate from diseases of the heart among Oregonians aged 15–34 years of 2.9 and 4.1 deaths per 100,000, during 2019 and 2021, respectively. Although the rate was higher during the pandemic year of 2021, myocarditis remained an infrequent cause of death among persons in this age group.^††^Detection of a small difference in mortality rate from myocarditis would require a larger sample size.

In this study of 1,292 deaths among Oregon residents aged 16–30 years during June 2021–December 2022, none could definitively be attributed to cardiac causes within 100 days of receipt of an mRNA COVID-19 vaccine dose; one male died from undetermined causes 45 days after receipt of a COVID-19 vaccine. During May 1, 2021–December 31, 2022, a total of 979,289 doses of COVID-19 vaccines were administered to Oregonians aged 16–30 years (unpublished data, ALERT IIS, 2024.)

During the same period, COVID-19 was cited as the cause of death for 30 Oregon residents in this age group. Among these 30 decedents, ALERT IIS had records for 22 (73%), only three of whom had received any COVID-19 vaccination. Studies have shown significant reductions in COVID-19–related mortality among vaccinated persons; during the first 2 years of COVID-19 vaccine availability in the United States, vaccination prevented an estimated 18.5 million hospitalizations and 3.2 million deaths (*9*).

### Limitations

The findings in this report are subject to at least two limitations. First, this report cannot exclude the possibility of vaccine-associated cardiac deaths >100 days after COVID-19 vaccine administration. However, published data indicate that potential adverse events associated with vaccinations tend to occur within 42 days of vaccine receipt (*10*). Second, small population size made it less likely that Oregon would see a rare event such as sudden cardiac death among adolescents and young adults.

### Implications for Public Health Practice

These data do not support an association between receipt of mRNA COVID-19 vaccine and sudden cardiac death among previously healthy young persons. COVID-19 vaccination is recommended for all persons aged ≥6 months to prevent COVID-19 and complications, including death.

## Footnotes

*
www.cdc.gov/vaccines/acip/work-groups-vast/report-2021-05-17.html

†
https://www.cdc.gov/vaccines/acip/meetings/downloads/slides-2021-06/04-COVID-Lee-508.pdf

§
https://www.nytimes.com/2022/01/28/technology/covid-vaccines-misinformation.html

¶
https://www.oregon.gov/oha/PH/BirthDeathCertificates/VitalStatistics/death/Pages/index.aspx

** 45 C.F.R. part 46.102(l)(2), 21 C.F.R. part 56; 42 U.S.C. Sect. 241(d); 5 U.S.C. Sect. 552a; 44 U.S.C. Sect. 3501 et seq.

†† https://wonder.cdc.gov/ucd-icd10-expanded.html (Accessed February 12, 2024).
